# Elevated STAT3 Signaling-Mediated Upregulation of MMP-2/9 Confers Enhanced Invasion Ability in Multidrug-Resistant Breast Cancer Cells

**DOI:** 10.3390/ijms161024772

**Published:** 2015-10-16

**Authors:** Fei Zhang, Zhiyong Wang, Yanling Fan, Qiao Xu, Wei Ji, Ran Tian, Ruifang Niu

**Affiliations:** 1 Public Laboratory, Tianjin Medical University Cancer Institute and Hospital, National Clinical Research Center for Cancer, Tianjin 300060, China; E-Mails: feizhang03@tmu.edu.cn (F.Z.); wzy7848354@hotmail.com (Z.W.); fanyanling402@163.com (Y.F.); xq691589132@hotmail.com (Q.X.); jiwei217@126.com (W.J.); tianrants1985@163.com (R.T.); 2 Key Laboratory of Breast Cancer Prevention and Therapy, Tianjin Medical University, Ministry of Education, Tianjin 300060, China; 3 Key Laboratory of Cancer Prevention and Therapy, Tianjin 300060, China

**Keywords:** STAT3, MMP-2, MMP-9, multidrug-resistant (MDR), invasion, breast cancer

## Abstract

The development of multidrug resistance greatly impedes effective cancer therapy. Recent advances in cancer research have demonstrated that acquisition of multidrug resistance by cancer cells is usually accompanied by enhanced cell invasiveness. Several lines of evidence indicated that cross activation of other signaling pathways during development of drug resistance may increase invasive potential of multidrug-resistant (MDR) cancer cells. However, the accurate mechanism of this process is largely undefined. In this study, to better understand the associated molecular pathways responsible for cancer progression induced by drug resistance, a MDR human breast cancer cell line SK-BR-3/EPR with P-glycoprotein overexpression was established using stepwise long-term exposure to increasing concentration of epirubicin. The SK-BR-3/EPR cell line exhibited decreased cell proliferative activity, but enhanced cell invasive capacity. We showed that the expression of metastasis-related matrix metalloproteinase (MMP)-2/9 was elevated in SK-BR-3/EPR cells. Moreover, SK-BR-3/EPR cells showed elevated activation of STAT3. Activation of STAT3 signaling is responsible for enhanced invasiveness of SK-BR-3/EPR cells through upregulation of MMP-2/9. STAT3 is a well-known oncogene and is frequently implicated in tumorigenesis and chemotherapeutic resistance. Our findings augment insight into the mechanism underlying the functional association between MDR and cancer invasiveness.

## 1. Introduction

Breast cancer remains the major cause of cancer death among females in spite of advances in breast cancer therapy in the past three decades [1]. Chemotherapy is one of the major strategies for treating this fatal disease, especially for advanced stage of breast cancer [2,3,4]. However, development of drug resistance greatly impedes effective cancer therapy [2,5,6,7]. More intractably, resistance to single chemotherapeutic drug by cancer cells are always associated with the emergence of cross-resistance to multiple structurally, functionally, and mechanistically unrelated anticancer drugs [2,5,6,7]. This phenomenon is known as multidrug resistance. Numerous studies have revealed several mechanisms that contribute to multi-drug resistance [2,5,6,7]. Overexpression of multidrug-efflux transporters, such as P-glycoprotein, MRP1, and BCRP, contributes to multidrug resistance [2,5,6,7,8]. These membrane transporters function as pumps that efflux cytotoxic reagents out of the cell, thereby resulting in drug resistance. Several studies have demonstrated that activation of cellular signaling contributes to upregulated expression of these drug transporters [9,10,11]. However, detailed mechanisms on how cancer cells upregulate these drug pumps and evolve the ability to resist apoptotic stimuli by anticancer drugs remain poorly understood.

Acquisition of drug resistance by cancer cells *in vitro* is usually accompanied by other changes of cellular activities, such as alterations in cell proliferative rate, resistance to apoptosis stimuli, and enhanced cell invasiveness [12,13,14,15,16,17,18,19]. Moreover, expression of metastasis-related genes is upregulated in many multidrug-resistant (MDR) cancer cells as compared to drug-sensitive cells [20,21,22,23]. In addition, MDR cancer cells even exhibit epithelial to mesenchymal transition signatures, which are associated with cancer invasion and metastasis [13,14,24,25,26,27]. Consistently, several *in vivo* studies found that MDR cancer cells displayed enhanced metastatic potential than parental cells in animal models [14,28]. Moreover, multidrug resistance also promotes tumor relapse and cancer metastasis clinically [21,22,29,30,31,32]. These results suggest a functional relationship between drug resistance and cancer cell invasion and metastasis. Thus, overcoming cancer progression caused by chemotherapy failure is urgently needed for cancer treatment. However, the accurate mechanism involved in this process is still largely unknown.

Several lines of evidence indicate that cross-activation of other signaling pathways during acquisition of drug resistance may increase the invasiveness potential of MDR cancer cells [9,10,23,33,34,35,36]. Establishing MDR cancer cell lines is highly important to improve the understanding of associated molecular pathways responsible for cancer progression induced by drug resistance. In the present study, a human breast cancer cell line SK-BR-3/EPR with MDR phenotype and overexpressing P-glycoprotein was established. In addition, we showed that SK-BR-3/EPR cells exhibited enhanced cell invasiveness along with upregulated expression of metastasis-related matrix metalloproteinase (MMP)-2/9. Moreover, SK-BR-3/EPR cells showed elevated activation of STAT3. We further provide evidence that activation of STAT3 signaling was responsible for enhanced invasiveness of SK-BR-3/EPR cells through upregulation of MMP-2/9. STAT3 is a well-known oncogene and frequently implicated in tumorigenesis and chemotherapeutic resistance. Therefore, our findings suggest a novel plausible mechanism employed by MDR cancer cells to promote their invasiveness.

## 2. Results

### 2.1. Establishment of Multidrug-Resistant (MDR) SK-BR-3/EPR Cells

**Figure 1:**
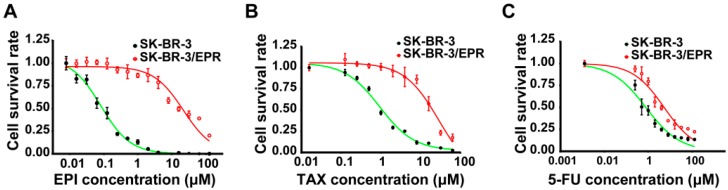
Establishment of a multi-drug resistant human breast cancer cell line SK-BR-3/EPR. (**A**–**C**) Drug sensitivity assay of SK-BR-3 and SK-BR-3/EPR cells to epirubicin, paclitaxel and 5-Fluorouracil (5-FU). Cell viability assay was determined using a Cell Counting Kit-8 (CCK-8) according to the manufacturer’s protocol. In brief, cells were seeded in 96 well plate at a density of 5 × 10^3^ cells per well and cultured for 24 h, then different concentration of indicated drugs were added into each well and further cultured for 72 h. After removal of culture medium, 10 μL of CCK-8 reagent in 200 μL medium were added into each well and incubated for 3 h, then the cell viability were calculated by measuring the absorbance at 450 nm on a micro-ELISA reader. The assays were performed using five replicates for each concentration and repeated three times. IC_50_ is defined as the concentration of drug causing half inhibition of cell growth compared with the cell growth of the control group. The IC_50_ was calculated by the Graphpad Prism 6.00 software.

SK-BR-3 is frequently used for studies on breast cancer cell biology and anticancer therapy, but induction of drug resistance in this well-known cell line has not been explored. An epirubicin-resistant cancer cell line was induced from parental SK-BR-3 cell lines by stepwise long-term exposure to increasing concentration of epirubicin over eight months. Epirubicin concentration was increased from 0.05 to 4.0 μM. The established resistant cells were designated as SK-BR-3/EPR (epirubicin resistant). As shown in Figure 1A and Table 1, the SK-BR-3/EPR cells were approximately 200-fold resistant to epirubicin than the parent cells. In addition, we also examined the sensitivity of these two cells to paclitaxel and 5-Fluorouracil (5-FU). As shown in Figure 1A,B, the SK-BR-3/EPR cells also exhibited a cross-resistant phenotype to these two chemotherapeutics drugs (Table 1), which they were not exposed to, suggesting it was a potential MDR cell line.

**Table 1:** IC_50_ (the concentration of drug causing half inhibition of cell growth compared with the cell growth of the control group) values in parental SK-BR-3 and SK-BR-3/EPR to various chemotherapeutic drugs.

Drugs	IC_50_ (μM)		*RI*	*p*-Value
	SK-BR-3	SK-BR-3/EPR		
Epirubicin	0.113 ± 0.0123	22.61 ± 1.258	199.4	<0.0001
Paclitaxel	1.372 ± 0.1050	23.50 ± 1.154	17.1	<0.0001
5-fluorouracil	1.080 ± 0.1661	5.81 ± 0.697	5.4	<0.0001

### 2.2. Elevated Expression of P-Glycoprotein Is Responsible for Acquisition of MDR Phenotype in SK-BR-3/EPR Cells

**Figure 2:**
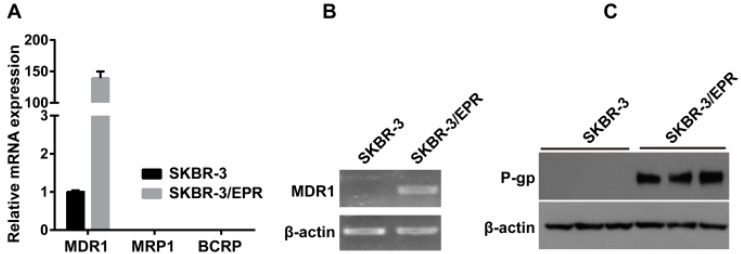

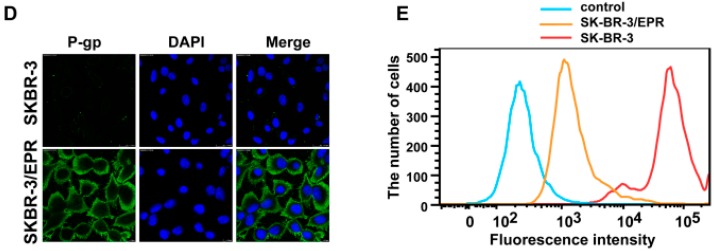
P-glycoprotein is upregulated in SK-BR-3/EPR cells. (**A**) Quantitative PCR analysis of expression of MDR1, MRP1, BCRP mRNA in SK-BR-3 and SK-BR-3 cells. The experiments were performed using three replicates for each group and repeated three times; (**B**) Further analysis of PCR products by agarose gel electrophoresis confirmed the upregulated expression of MDR1 mRNA in SK-BR-3/EPR cells; (**C**) Western blotting analysis of P-glycoprotein expression in SK-BR-3 and SK-BR-3/EPR cells; (**D**) Confocal immunofluorescence microscopy analysis showed positive expression of P-glycoprotein in SK-BR-3/EPR cells, but not in drug sensitive SK-BR-3 cells (×600 magnification); (**E**) SK-BR-3/EPR cells showed markedly reduced retention of intracellular Rh123 compared with SK-BR-3 cells as measured by rhodamine efflux assay.

Upregulation of drug transporters, namely, MDR1/P-glycoprotein, MRP1, and BCRP is well-known to confer to multidrug resistance [6]. We investigated the expression patterns of MDR1, MRP1 and BCRP mRNA in SK-BR-3, as well as SK-BR-3/EPR, by quantitative PCR. As shown in Figure 2A, the expression of MDR1 mRNA in SK-BR-3/EPR cells was markedly upregulated approximately 150-fold than that in the parent cells, whereas the mRNA expression of MRP1 and BCRP was not detected in both cells. Further analysis of PCR products by agarose gel electrophoresis confirmed the upregulated expression of MDR1 mRNA in SK-BR-3/EPR cells (Figure 2B). Consistently, data from Western blotting showed strong positive expression of P-glycoprotein in SK-BR-3/EPR cells, while, P-glycoprotein was undetected in parent SK-BR-3 cells (Figure 2C). In addition, confocal immunofluorescence microscopy analysis also confirmed the apparent expression of P-glycoprotein in the cell membrane of SK-BR-3/EPR cells, but not in drug sensitive cells (Figure 2D). Moreover, SK-BR-3/EPR cells exhibited markedly reduced retention of intracellular rhodamine 123 dye compared with SK-BR-3 cells as measured by rhodamine efflux assay (Figure 2E). This phenomenon is also correlated with P-glycoprotein expression and function

### 2.3. SK-BR-3/EPR Cells Maintained the MDR Phenotype after Sequential Passages in Epirubicin-Free Medium for Six Weeks

**Figure 3:**
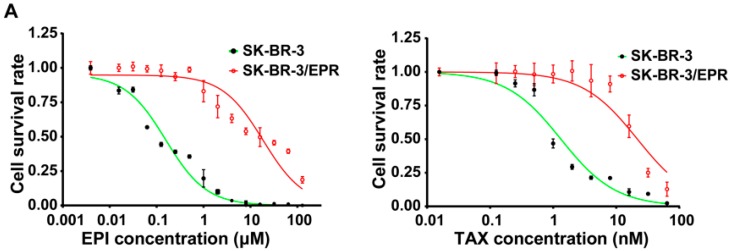

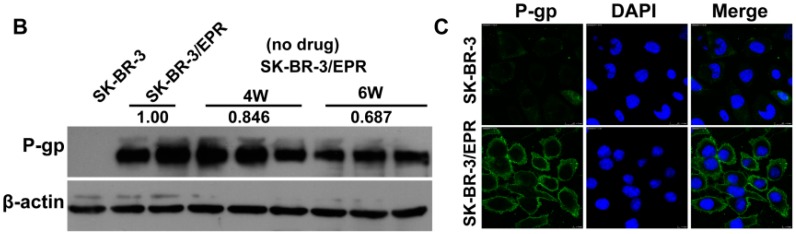
The SK-BR-3/EPR cells maintained MDR phenotype after sequential passages in epirubicin-free medium for six weeks. (**A**) Drug sensitivity assay of SK-BR-3 and SK-BR-3/EPR cells to epirubicin and paclitaxel. The SK-BR-3/EPR cells were cultured in epirubicin-free medium and sequential passages for six-consecutive weeks, then IC_50_ assay was performed by using the CCK-8 based method; (**B**) Western blotting analysis of P-glycoprotein expression (P-gp) in SK-BR-3, SK-BR-3/EPR, and SK-BR-3/EPR cells cultured in drug-free condition for indicated times. The numbers above the blots show the relative values from the densitometric analysis. The levels of P-gp was normalized to β-actin, values are expressed as fold changes compared to SK-BR-3/EPR cells; (**C**) Confocal immunofluorescence microscopy analysis of P-glycoprotein expression in SK-BR-3 and SK-BR-3/EPR cells cultured in epirubicin-free medium for six-consecutive weeks (×600 magnification).

Long-term persistence of the MDR phenotype in SK-BR-3/EPR cells under drug-free conditions was investigated. The SK-BR-3/EPR cells were cultured in epirubicin-free condition for six consecutive weeks. Then, the cells resistant to epirubicin and paclitaxel were examined. As shown in Figure 3A,B and Table 2, the SK-BR-3/EPR cells displayed evident resistance to the two tested drugs after sequential passages in epirubicin-free medium for six weeks. Western blot and immunofluorescence results showed positive expression of P-glycoprotein in SK-BR-3/EPR cells compared with that in the parent SK-BR-3 cells (Figure 3B,C). Thus, these data suggest that the SK-BR-3/EPR cells could sustain the MDR phenotype in a drug-free condition.

**Table 2:** IC_50_ values in SK-BR-3 and SK-BR-3/EPR cells after sequential passages in epirubicin-free medium for six weeks.

Drugs	IC_50_ (μM)		*RI*	*p*-Value
	SK-BR-3	SK-BR-3/EPR		
Epirubicin	0.139 ± 0.0151	21.28 ± 2.899	153.1	<0.0001
Paclitaxel	1.416 ± 0.1542	20.87 ± 0.560	14.7	<0.0001

### 2.4. The SK-BR-3/EPR Cells Displayed a Marked Reduction in Cell Proliferation Rate, and Enhancement in Cell Invasion Ability

**Figure 4:**
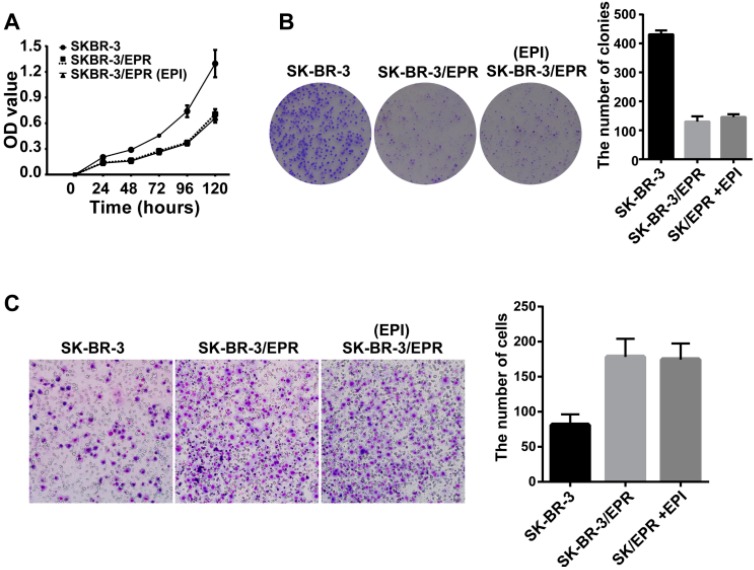
The SK-BR-3/EPR cells exhibited a marked reduction in cell proliferation rate, and enhancement in cell invasion ability. (**A**) The SK-BR-3/EPR cells showed decreased cell proliferation rate in the presence or absence of epirubicin in comparison with that of drug sensitive SK-BR-3 cells; (**B**) The SK-BR-3/EPR cells showed a remarkably reduction in the formation of cell colonies compared with that of in drug sensitive SK-BR-3 cells. Cells were seeded in 35-mm dishes at a density of 500 cells per dish and then cultured for 10–14 days, the number of colonies was counted under an inverted microscope, and only colonies of more than 50 individual cells were counted (×20 magnification). The assays were performed in triplicate for each group and repeated three times; (**C**) The SK-BR-3/EPR cells exhibited enhanced invasive ability compared with that of drug sensitive SK-BR-3 cells. The cells were cultured to 80% confluence, and harvested by trypsinization, then the cell suspension at a density of 4 × 105 cells/mL were added into the matrigel pre-coated transwell insert. The lower insert was filled with 10% FBS-containing medium. After incubation for 24 h at 37 °C, the invaded cells were fixed, stained, and counted. The assay was performed in triplicate and repeated three times (×200 magnification).

The cell proliferative potential of SK-BR-3/EPR and its parent cells was also examined using the CCK-8 based assay. As shown in Figure 4A, SK-BR-3/EPR cells displayed a significantly reduced in cell proliferation ability in the absence or presence of epirubicin. Similarly, SK-BR-3/EPR cells also showed remarkably reduced formation of cell colonies compared with that of drug sensitive SK-BR-3 cells, as measured by colony formation assay (Figure 4B). We then compared the invasive potential between SK-BR-3/EPR and its parental cells using Transwell-based assay. Interestingly, the number of SK-BR-3/EPR cells that invade through the Matrigel-coated membrane was two-fold greater than that of SK-BR-3 cells, thereby indicating the enhanced invasiveness of SK-BR-3/EPR cells (Figure 4C).

### 2.5. Expression of MMP-2/9 Was Upregulated in SK-BR-3/EPR Cells

**Figure 5:**
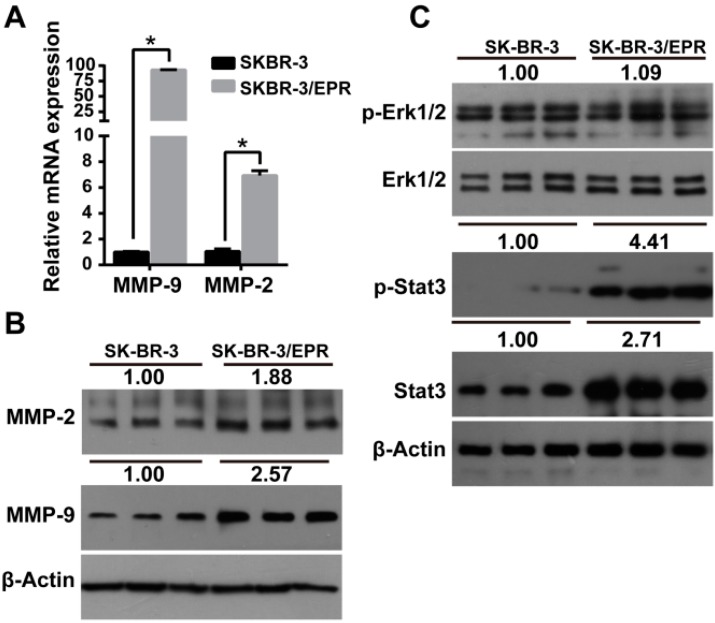
Expression of MMP-2 and -9 as well as phosphorylation of STAT3 was elevated in SK-BR-3/EPR cells. (**A**) Quantitative PCR analysis of expression of MMP-2 and -9 mRNA in SK-BR-3 and SK-BR-3/EPR cells. The experiments were performed using three replicates for each group and repeated three times; (**B**) Western blotting analysis of expression of MMP-2 and -9 protein in SK-BR-3 and SK-BR-3/EPR cells. The numbers above the blots show the relative values from the densitometric analysis. The levels of MMP-2 and -9 were normalized to β-actin, values are expressed as fold changes compared to SK-BR-3 cells; (**C**) Phosphorylation and activation of STAT3 was increased in MDR SK-BR-3/EPR cells. Western blotting analysis of expression of phosphorylation of Erk1/2 and STAT3 in SK-BR-3 and SK-BR-3/EPR cells. SK-BR-3 and SK-BR-3/EPR cells were grown to 90% confluence, then total cellular proteins were extracted and subjected to Western blotting analysis. The numbers above the blots show the relative values from the densitometric analysis. The level of Stat3 was normalized to β-actin, p-Erk1/2 and p-Stat3 were normalized to the corresponding total Erk1/2 and Stat3, values are expressed as fold changes compared to SK-BR-3 cells. * *p* < 0.05.

Invasion processes require upregulation of proteolytic enzymes, such as MMP-2/9, to degrade extracellular matrix, thereby facilitating the invasion of cancer cells to surrounding tissues [29,37,38,39]. We then determined the expression levels of MMP-2/9 in these two cells using quantitative PCR and Western blotting methods. As shown in Figure 5A, the expression levels of MMP-2/9 mRNA were significantly upregulated in SK-BR-3/EPR cells than SK-BR-3 cells. Consistently, the protein levels of MMP-2/9 was significantly upregulated in MDR SK-BR-3/EPR cells (Figure 5B).

### 2.6. Phosphorylation and Activation of STAT3 Was Increased in MDR SK-BR-3/EPR Cells

Previous studies have demonstrated that activation of Erk1/2 and STAT3 signaling is involved in the upregulation of the expression of MMP-2/9 [20,38,39,40]. Therefore, the expression and phosphorylation of STAT3 and Erk1/2 in the parental and SK-BR-3/EPR cell lines were examined by Western blot analysis. Figure 5C showed that no significant difference of Erk1/2 and its phosphorylation was observed between these two cells. However, phosphorylation of STAT3 was remarkably increased in SK-BR-3/EPR cells compared with its parental SK-BR-3 cells (Figure 5C), which indicated that the activation of STAT3 was increased in SK-BR-3/EPR cells compared with that in the parental cells.

### 2.7. Knockdown of STAT3 Inhibited Cell Invasion and Downregulated the Expression of MMP-2/9 in SK-BR-3/EPR Cells

**Figure 6:**
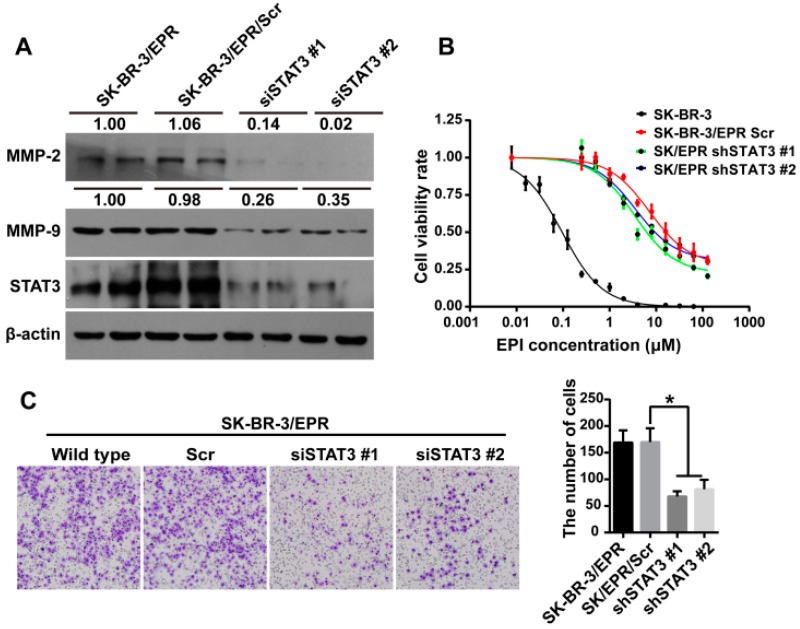
Knockdown of STAT3 inhibited cell invasion and downregulated expression of MMP-2 and -9 in SK-BR-3/EPR cells. (**A**) Knockdown of STAT3 significantly reduced the expression levels of MMP-2 and -9 protein in SK-BR-3/EPR cells. Western blotting analysis of STAT3, MMP-2, and -9 expression in SK-BR-3/EPR cells and SK-BR-3/EPR cells transfected with control or STAT3 specific siRNAs. The numbers above the blots show the relative values from the densitometric analysis. The levels of MMP-2 and -9 were normalized to β-actin, values are expressed as fold changes compared to SK-BR-3/EPR cells; (**B**) Knockdown of STAT3 in SK-BR-3/EPR cells slightly increased the sensitivity to epirubicin in comparison with control cells; (**C**) Knockdown of STAT3 remarkably inhibited the invasive ability of SK-BR-3/EPR cells as measured by transwell based assay (×200 magnification). * *p* < 0.05.

To further investigate whether the elevated activation of STAT3 contributes to the enhancement in cell invasiveness of SK-BR-3/EPR cells, STAT3 expression was downregulated using STAT3 specific siRNA. Figure 6A showed that the expression levels of MMP-2/9 was significantly downregulated in STAT3 knockdown cells compared with control cells and non-transfectedSK-BR-3/EPR cells. Then, the effect of STAT3 knockdown on cell invasion ability was analyzed using Transwell-based assay. As shown in Figure 6C, the amount of cells that invaded through the membrane to the lower chamber in STAT3-silenced group was significantly reduced compared with that in the control group. Moreover, the sensitivity to epirubicin in STAT3 silenced cells was also investigated, as shown in Figure 6B and Table 3, the IC_50_ value of epirubicin in STAT3 silenced cells was slightly reduced compared with that of control cells. This phenomenon indicated that activation of STAT3 is partially responsible for elevated drug resistance in SK-BR-3/EPR cells.

**Table 3:** IC_50_ of epirubicin in SK-BR-3 and SK-BR-3/EPR cells transfected with control or STAT3 specific siRNAs.

Cell Type	IC_50_ (μM)	*RI*	*p*-Value
SK-BR-3	0.127 ± 0.0289	-	-
SK-BR-3/EPR Scr	19.42 ± 0.0483	152.9	<0.0001
SK-BR-3/EPR shSTAT3 #1	7.913 ± 0.0574	62.3	<0.0001
SK-BR-3/EPR shSTAT3 #2	12.87 ± 0.0605	101.3	<0.0001

## 3. Discussion

Chemotherapy is the major treatment of most metastatic solid tumors, especially for advanced breast cancer [2,3,4]. However, resistance to chemotherapeutic drugs greatly impedes effective treatment of this fatal disease [5,6,7,8]. Recent advances in anticancer research revealed that acquisition of drug resistance by cancer cells not only leads to treatment failure, but also promotes cancer progression, such as more rapid tumor relapse and enhancement in cancer cell invasion and metastasis [32]. Thus, defining the accurate mechanism involved in the development of drug resistance and associated molecular pathways responsible for cancer progression induced by drug resistance is urgent for anticancer therapy. In the present study, a novel MDR breast cancer cell line was successfully established. Cellular function analysis demonstrated that SK-BR-3/EPR cell line exhibited reduced proliferative activity, but enhanced cell invasiveness. Therefore, this work provides a novel model for studying enhanced invasiveness of MDR cancer cells. Moreover, the expression of MMP-2 and -9 was upregulated in the established SK-BR-3/EPR cell line. The elevated phosphorylation and activation of STAT3 is responsible for the enhanced invasive properties and upregulated expression of MMP-2 and -9 in SK-BR-3/EPR breast cancer cells. Thus, our findings suggest a novel mechanism employed by drug-resistant breast cancer cells to promote cell invasion capacity.

Anthracyclines, such as epirubicin and adriamycin, are major chemotherapeutic agents for breast cancer chemotherapy [8]. However, resistance to these drugs often develops cross resistance of cells to other drugs that the cells have not been exposed to, leads to MDR phenotype [5,6,7,8]. Overcoming multidrug resistance is certainly beneficial for cancer treatment to improve the prognosis of cancer patients. However, acquisition of MDR is a very complex process, such that cancer cells can evolve several mechanisms to against different cytotoxic drugs [5,6,7,8]. Therefore, further elucidating the molecular process during the acquirement of MDR phenotype is essential. The establishment of drug-resistant cell lines is critical for investigating the mechanisms of MDR and exploring approaches for reversal of resistance. In this study, a stable epirubicin-resistant SK-BR-3 cell line with P-glycoprotein overexpression was successfully established. In addition to epirubicin resistance, the SK-BR-3/EPR cells also exhibited cross resistance to paclitaxel and 5-FU, indicating that this cell line was an MDR cell line. P-glycoprotein is important in mediating drug efflux. Thus, our study provides a new cell line for further exploration of the mechanism of drug resistance induced by P-glycoprotein overexpression. To our knowledge, this paper is the first report of the establishment of epirubicin-resistant SK-BR-3 cell line with MDR phenotype and P-glycoprotein overexpression.

Evolution of chemotherapy resistance of cancer cells has been associated with elevated cell invasiveness *in vitro* [17]. MDR cancer cells also displayed enhanced metastatic potential in animal models [14,28]. In the clinic, MDR in cancer patients is always correlated with more rapid tumor recurrence and metastasis [21,22,29,30,31,32]. However, the mechanism by which drug-resistant cancer cells acquire enhanced invasiveness, in addition to their acquired MDR phenotype, remains poorly understood. Thus, clarifying the mechanism for cancer cell invasiveness induced by drug resistance is urgent for improving cancer treatment. In this study, we found that the mRNA and protein levels of MMP-2/9 was significantly upregulated in SK-BR-3/EPR cells compared with that in the parental SK-BR-3 cells. This result is consistent with findings of previous reports in which MDR cancer cells displayed increased production of MMP-2 or MMP-9 [23,41,42]. MMPs are key proteolytic enzymes for degradation of the extracellular matrix by cancer cells. These results indicated that upregulation of MMP-2/9 may be a mechanism for enhanced cell invasion of SK-BR-3/EPR cells.

The mechanism by which MDR cancer cells upregulate MMPs and promote their invasiveness is very worthy of investigation. The expression of MMP-2/9 is reported to be regulated by Erk1/2 signaling [20,38,39,41,43]. Previous studies have demonstrated that several MDR cancer cells exhibited increased phosphorylation of Erk1/2 compared with drug-sensitive cells [20,41,44,45]. However, in our study, the phosphorylation of Erk1/2 was not significantly altered in SK-BR-3/EPR cells compared with that in drug-sensitive SK-BR-3 cells. Thus, the upregulated expression of MMP-2/9 may not be regulated by Erk1/2 signaling in this cell model. In addition to Erk1/2 signaling, other studies have shown that the expression of MMP-2/9 is also regulated by the STAT3 pathway [38,39,46,47], and activation of STAT3 is also associated with drug resistance of cancer cells [48,49,50]. Here, we showed that the phosphorylation of STAT3 as well as its nuclear translocation, which indicating the activation of STAT3, was remarkably increased in SK-BR-3/EPR cells. Moreover, we further showed that silencing of STAT3 through siRNA significantly downregulated the protein levels of MMP-2/9. This phenomenon suggested that elevated activation of STAT3 is responsible for the upregulation of MMP-2 and -9 in SK-BR-3/EPR cells. Depletion of STAT3 also inhibited invasiveness of MDR SK-BR-3/EPR cells. Hence, activation of STAT3 signaling is responsible for the enhanced cell invasiveness via upregulation of MMP-2/9 expression.

In summary, an epirubicin-resistant cell line SK-BR-3/EPR with P-glycoprotein overexpression was established in this study. The cell line showed MDR phenotype and decreased cell proliferative activity, but enhanced cell invasiveness. We also showed that the expression of MMP-2/9 was upregulated in SK-BR-3/EPR cells. Moreover, we provided evidence that elevated STAT3 signaling mediates upregulation of MMP-2/9 and confers increased invasiveness in SK-BR-3/EPR breast cancer cells. Thus, our findings augment insight into the mechanism underlying the functional association between MDR and cancer invasiveness.

## 4. Experimental Section

### 4.1. Reagents and Drugs

RPMI 1640 medium and Trypsin were obtained from Hyclone (Logan, UT, USA). Fetal bovine serum (FBS) was obtained from Gibco (Carlsbad, CA, USA). Epirubicin was obtained from Hisunpharm Co. (Taizhou, Zhejiang, China). Cell Counting Kit-8 (CCK-8) was purchased from Dojindo Laboratories (Kumamoto, Japan). M-MLV reverse transcriptase and SYBR real-time PCR kit were obtained from Takara (Dalian, China). Trizol reagent and Lipofectamine 2000 was purchased from Invitrogen (Carlsbad, CA, USA). Transwell inserts were obtained from Millipore (Darmstadt, Germany). Protease inhibitor (EDTA-free) were obtained from Roche Diagnostics (Mannheim, Germany).Rabbit polyclonal antibodies against P-glycoprotein, MRP1 and mouse monoclonal antibodies against β-actin was obtained from Santa Cruz Inc. (Santa Cruz, CA, USA). Rabbit monoclonal antibodies against STAT3, MMP-2, and MMP-9 were purchased from Epitomics (Burlingame, CA, USA). Rabbit monoclonal antibodies against Erk1/2, p-Erk1/2 (Thr202/Tyr204), and p-STAT3 (Tyr 705) were purchased from Cell Signaling Technology (Danvers, MA, USA). Mouse monoclonal antibody against BCRP was purchased from Abcam (Cambridge, UK). Enhanced chemiluminescence reagents (ECL) were purchased from Millipore (Darmstadt, Germany).

### 4.2. Cell Culture and Induction of Drug Resistance

Human breast cancer cell lines SK-BR-3 were obtained from the American Type Culture Collection (Manassas, Virginia, USA). Cells were cultured in RPMI-1640 culture medium containing 10% FBS in an incubator humidified at 37 °C with 5% CO_2_. For induction of drug resistance cell line, SK-BR-3 cells were cultured in 6 cm dishes at a density of approximately 20% confluence. The cells were initially exposed to approximately 25% of IC_50_ doses of epirubicin (0.025 µM). The medium was changed every two days. Survival cells were continuously exposed to 0.025 µM epirubicin until the cells could grow steadily at this drug concentration. Then, the cells were exposed to epirubicin at 1.5- to 2-fold increase from the previous concentration. After 8 months induction, the cells grew stably in the presence of 4.0 µM of epirubicin. Subsequently, the cells were continuously exposed to 4.0 µM of epirubicin for 2 months. The generated resistant cell line was designated as SK-BR-3/EPR (epirubicin resistant). Before experimentation, he SK-BR-3/EPR cells were cultured in drug-free medium for at least 2 weeks.

### 4.3. IC_50_ Assay

IC_50_ assay was evaluated using CCK-8-based method (according to the manufacturer’s instruction). SK-BR-3/EPR cells were cultured in drug-free condition for 2 weeks. Then SK-BR-3/EPR and parental cells were cultured in 96-well plate at a density of 5 × 10^3^ cells per well. After 24 h of incubation, different concentrations of drugs were added into cells and further incubated for 72 h. Cell viability was evaluated at 450 nm on a micro-ELISA reader. IC_50_ is defined as the concentration of drug causing half inhibition of cell growth compared with the cell growth of the control group. Assays were carried out using five replicates for each concentration and repeated thrice. IC_50_ was calculated by the Graphpad Prism 6.00 software (Graphpad Software, La Jolla, CA, USA). The ratio between IC_50_ values of the MDR cancer cells and the sensitive cells was defined as the resistance index (*RI*). *RI* values ≥ 3 indicated chemoresistant cancer cell line.

### 4.4. Reverse Transcription and Quantitative PCR

Reverse Transcription and Quantitative PCR was carried out as described previously [17]. Briefly, total cellular RNA was extracted using the Trizol reagent, and 1 µg of total RNA was reverse-transcribed to complementary DNA (cDNA) using the M-MLV RT kit. Then the cDNA was used for quantitative real-time PCR analysis using the SYBR Premix Ex Taq according to the manufacturer’s protocol. The PCR Primers are as follows: MDR1 (upper: 5′-TCCTGGAGCGGTTCTACGAC-3′, lower: 5′-ATGGGCTCCTGGGACACGAT-3′), MRP1 (upper: 5′-GGAACTACTGCCTGCGCTAC-3′, lower: 5′-TCTCCTTCGGCAGACTCGTT-3′), BCRP (upper: 5′-AGTACTTCAGCATTCCACGAT-3′, lower: 5′-TCGATGCCCTGCTTTACCAA-3′), β-Actin (upper: 5′-CAGAGCAAGAGAGGCATCC-3′, lower: 5′-CTGGGGTGTTGAAGGTCTC-3′). The assays were conducted using three replicates for each cell line and repeated thrice.

### 4.5. Western Blotting

Western blotting assay was carried out as described previously [51]. In brief, cells were cultured to 90% confluence in 6 cm dishes, then cells were washed with PBS and total cellular protein was extracted using 400 μL of 1× SDS cell lysis buffer (50 mM Tris, 150 mM NaCl, 2% SDS, 10% glycerol, 5% 2-mercaptoethanol, and 1× protease inhibitor cocktail at pH 6.8). The lysates were quantified, and 20 μg of cell lysates was resolved on SDS-PAGE and transferred onto PVDF membranes. The membrane was blocked with 5% non-fat milk in TBST buffer at room temperature for 1 h, and then probed with corresponding primary antibodies overnight at 4 °C. Followed by incubation with corresponding horseradish peroxidase (HRP)-linked secondary antibody, and then detected using an ECL reagents according to the manufacturer’s instructions. β-actin was used as a loading control.

### 4.6. Immunofluorescence Assay

Immunofluorescence assay was carried out as described previously [52]. Briefly, cells were seeded on glass coverslips at a density of 5 × 10^4^ cells/mL in 12 well plates and incubation for 24 h. Then the cells were washed with PBS, fixed with freshly prepared 4% PFA/PBS at RT for 10 min. After permeabilization in 0.2% triton X-100 for 10 min, the cells were blocked in 3% BSA at RT for 1 h, and probed with the indicated primary antibodies at 4 °C overnight. The cells were then stained with corresponding Alexa Fluor 488 and 594-conjugated secondary antibodies at RT for 1 h, followed by staining nucleus by using 1 ng/mL of DAPI. The coverslips were finally mounted with Mowiol-based anti-fading medium and visualized under a laser scanning confocal microscope (Leica TCS SP5, Leica Microsystems, Wetzlar, Hesse-Darmstadt, Germany).

### 4.7. Rhodamine 123 (R123) Dye Efflux Assay

R123 efflux assay was carried out as described previously [17]. Briefly, cells were cultured to 90% confluence in 6-cm dishes. The cells were harvested with trypsin, washed twice with PBS, and adjusted to a density of 5 × 10^5^ cells/mL. Then, Rh123 dye was added into the cell suspension to reach final concentration of 2 µg/mL. After incubation for 30 min at 37 °C, cells were washed twice with PBS, resuspended with 500 µL PBS and further incubated in Rh123-free medium for 10 min at 37 °C. After another twice washes with PBS, the mean fluorescence intensity (MFI) was immediately analyzed using flow cytometric analysis. The excitation and emission wavelength is 488 and 530 nm respectively. The assay was carried out in triplicate and repeated thrice.

### 4.8. Cell Proliferation Assay/Colony Formation Assay

Cell proliferative activity was evaluated using CCK-8 and colony formation assay. For CCK-8 assay, cells were cultured in 96-well plate at a concentration of 2 × 10^3^ cells per well and incubated for 24, 48, 72, 96, and 120 h. At each time point, each well was added with 10 μL of CCK-8 reagent, and further incubated for 3 h at 37 °C. The cell proliferative activity was calculated by a micro-ELISA reader using a filter for 450 nm. The assays were carried out using five replicates for each time group and repeated thrice. Colony formation assay was carried out as described previously [53]. In brief, 500 cells were seeded in 35-mm dishes and cultured for 10–14 days. After washing thrice with PBS, the cells were fixed with ice-cold methanol at 4 °C, and stained using a three-Step Stain Set kit (Thermo Scientific, Kalamazoo, MI, USA). The colonies containing more than 50 individual cells was counted. The assay was carried out in triplicate.

### 4.9. Cell Invasion Assay

Cell invasion ability was evaluated using a transwell-based assay as described previously [17]. Briefly, exponentially growing cells were harvested by trypsin digestion, washed thrice with PBS, resuspended in FBS-free medium and adjusted to a density of 4 × 10^5^ cells/mL. Then, 200 μL of cells (8 × 10^4^ cells) were added into the transwell insert (8 μm pore), which were pre-coated with matrigel. The lower insert was filled with 10% FBS-containing medium. After subsequent incubation for 24 h at 37 °C, the invaded cells were fixed with ice-cold ethanol, and stained using a three-Step Stain Set kit. The numbers of invaded cells were calculated under a light microscope. The experiment was carried out in triplicate and repeated thrice.

### 4.10. Statistical Analysis

All quantitative data were expressed as mean ± SD. Statistical analysis was conducted by one-way ANOVA using the Graphpad Prim 6.00 software. A *p* value less than 0.05 (two-tailed) was considered as significant. The asterisk indicates a *p* value <0.05.

## 5. Conclusions

An MDR breast cancer cell line SK-BR-3/EPR with P-glycoprotein overexpression was established in this study. SK-BR-3/EPR cells exhibited enhanced cell invasiveness and upregulation of MMP-2/9. Moreover, we showed that elevated STAT3 signaling-mediated upregulation of MMP-2/9 is responsible for the enhanced invasive properties in SK-BR-3/EPR breast cancer cells.
